# Bibliometric Mapping of Literature on High-Entropy/Multicomponent Alloys and Systematic Review of Emerging Applications

**DOI:** 10.3390/e24030329

**Published:** 2022-02-24

**Authors:** Akeem Damilola Akinwekomi, Farid Akhtar

**Affiliations:** Division of Materials Science, Luleå University of Technology, 97187 Luleå, Sweden; akeem.akinwekomi@ltu.se

**Keywords:** bibliometric mapping, high-entropy alloy, multicomponent alloy, text-mining, VOSviewer

## Abstract

High-entropy/multicomponent alloy (HEA/MCA) has received significant research attention in the last decade. There is a dearth of data-driven works dedicated to assessing and visualizing the HEA/MCA literature from a global perspective. To this end, we present the first bibliometric literature analysis of more than 3500 HEA/MCA articles, published between 2004 and 2021, in the Scopus database. We identify the most prolific authors, their collaborators, institutions, and most prominent research outlet. Co-occurrence networks of keywords are mapped and analyzed. A steep rise in research outputs is observed from 2013, when the number of annual publications doubled the previous years. The top five preferred research outlets include *Journal of Alloys and Compounds*, *Materials Science and Engineering A*, *Scripta Materialia, Intermetallics*, and *Acta Materialia*. Most of these publications emanate from researchers and institutions within China, USA, and Germany, although international scientific collaboration among them is lacking. Research gaps and future research directions are proposed, based on co-occurrence frequencies of author keywords. Finally, a brief systematic review of emerging applications, covering hydrogen storage, additive manufacturing, catalysis, and superconductivity, is undertaken. This work provides an important comprehensive reference guide for researchers to deepen their knowledge of the field and pursue new research directions.

## 1. Introduction

Conventional alloys are developed predominantly from one principal element, with other minor alloying elements, to impact primary and secondary properties, respectively [1]. It was previously hypothesized that the formation of glassy phases [1] or complex, brittle intermetallic compounds [2] is favored in alloy systems comprising of multi-principal or multicomponent elements. For instance, the propensity for the formation of an intermetallic compound is favored when the solvent and solute atoms have different atomic sizes, valence, and electronegativity, according to the classical Hume-Rothery rules [2,3]. In the past few years, solid-solution alloys with simple crystal structures from multi-principal metallic components (≥5) in near-equimolar or equimolar ratios have been reported [1,4,5,6]. It is proposed that the contribution of high mixing configurational entropy to the total free energy of the alloy can stabilize simple, solid-solution phases and suppress the formation of complex, intermetallic compounds [4,7]. These multi-component alloys may have complex compositions, but they are characterized by simple crystal structures. They have come to be known as high-entropy alloys (HEA) [4], multicomponent alloys (MCA) [1,8], or compositionally complex alloys [9].

Currently, there is no consensus on the strict definition of HEA or MCA [10,11]. It is generally acceptable to define them based on either their composition or entropy. Therefore, the compositional definition is given as an alloy with five or more principal elements, whose composition varies between 5 and 35 atomic weight percent (at.%) [4,9,12]. The other definition, based on configurational mixing entropy (*S*), gives a range of *S* ≥ 1.61*R* (where *R* is the standard gas constant) for an alloy with five principal elements in equimolar concentration [11]. This, however, may be extended to non-equimolar concentrations, provided the concentration of each element is between 5 and 35 at.% [4].

Ever since the publication of the first papers on HEAs [2,4,13] and MCAs [1], research interest in this field has been growing steadily. This has largely been spurred by the opportunity to explore new alloy compositions that transcend the scope of traditional alloy compositions, as well as the potential to investigate interesting new advanced applications. Besides, various research directions have been explored, including investigating the formation of simple crystalline phases, with the different number of principal elements (e.g., vanadium (V), hafnium (Hf), chromium (Cr), manganese (Mn), iron (Fe), copper (Cu), and nickel (Ni)), ranging from five to as many as twenty [1,2,5,6,14,15,16]. Similarly, researchers have utilized low-density metals, such as magnesium (Mg), aluminum (Al), lithium (Li), silicon (Si), titanium (Ti), and zinc (Zn), to develop lightweight structural HEA materials [17,18] for potential applications in the aerospace and transportation industries. Likewise, there are reports on the development of new, high-temperature structural materials, from refractory metals (e.g., niobium (Nb), zirconium (Zr), tungsten (W), etc.) [19,20,21] to HEA/MCA thin films and coatings [2,22,23]. While most of the literature in this field has been devoted to investigating different mechanical properties [22,23,24,25,26,27,28,29], a few have been targeted at corrosion/electrochemical studies [30,31], catalysis [32,33,34], superconductivity [35,36], magnetism [37,38], and thermo-electric properties [39].

With a large number of articles in this field, it may be challenging to comprehend the vast knowledge and keep abreast of new information. To this end, some excellent systematic review works, on key findings and future research directions, have been undertaken [3,11,30,40]. However, no bibliometric analysis of the HEA/MCA is available. A bibliometric analysis study (or science mapping) is a mechanistic approach that is based on text mining techniques, which can be applied to analyze and visualize different types of bibliometric networks, based on the outputs of the academic literature in a particular scientific field [41]. These may include analyzing networks of citations relationship between authors, journals, countries, co-authorship analysis of authors, or co-occurrence of keywords [41]. Other than visualization analyses, a bibliometric review helps to understand historical and evolutionary changes in a research field [42], foster a better understanding of the subject area, and create a comprehensive reference platform for future researchers. Typical examples of the application of bibliometric review include computational intelligence [43], library and information science [42], microbial fuel cell [44], and conversion technologies for carbon dioxide [45]. However, no such global review can be found on HEA/MCA materials.

Therefore, this present work employs a data-driven approach and visualization techniques to analyze the HEA/MCA literature, with the aim of providing a holistic science mapping of this field. This is achieved by bibliometrically reviewing the existing literature on HEA/MCA, from 2004 to May 2021, using the VOSviewer software [46]. The main objectives of this study are to: (i) identify the most active researchers and institutions, (ii) create a visual landscape of active countries in this field, (iii) analyze keyword co-occurrence and its associated network, (iv) assess high impact research outlets publishing the works in this field, and (v) conduct a systematic review of some emerging applications to identify some potential future research directions. Therefore, this paper will offer useful insight for fostering potential research collaborations and suggest potential future research directions.

## 2. Materials and Methods

### 2.1. Data Mining Strategy

All bibliometric data were extracted from the Scopus database. A query string containing keywords was used to search in the title, abstract, and keyword fields. Since the first publications on HEA/MCA were published in 2004, in English, the query string was refined to exclude articles published before this year, as well as those in a language other than English. The following string retrieved more than 3500 items as of 25 April 2021: (TITLE-ABS-KEY(“high entropy alloy*” OR “high-entropy alloy*” OR “multicomponent alloy*” OR “multiprincipal element alloy*” OR “equiatomic multicomponent” OR “composit* complex alloy*”) AND NOT TITLE-ABS-KEY(“binary” OR “steel” OR “equilibrated” OR “binary alloy” OR “ternary” OR “equivalent” OR “super alloy*” OR “superalloy” OR “Inconel”) AND NOT TITLE(“glass*” OR “glass-form*”)) AND PUBYEAR > 2003 AND (LIMIT-TO (DOCTYPE, “ar”)) AND (LIMIT-TO (LANGUAGE, “English”)) AND (LIMIT-TO (SRCTYPE, “j”)). These articles were subjected to further text cleaning and bibliometric analyses.

### 2.2. Text Cleaning

Before statistically analyzing the retrieved articles, their abstract, title, and keywords were manually screened to detect and remove any irrelevant ones. For instance, the keyword “multicomponent alloy*” retrieved many articles, such as [47,48,49,50,51,52], which did not belong to the field of HEA. Eventually, 3541 research articles were retrieved and analyzed. Then, text cleaning was undertaken to unify various forms of some author keywords. For instance, keywords such as “high-entropy”, “HEA”, “high entropy” were combined and unified as “high-entropy”. Similarly, all variants of the keywords “multi-component”, “single phase FCC”, “concentrated alloys, concentrated solid-solution alloys, complex concentrated alloy”, and “multiple principal element alloy” were combined and unified as “multi-component”, “FCC”, “compositionally complex alloy”, and “multi-principal element alloy”, respectively. Text cleaning was accomplished using v. 3.4.1 of OpenRefine (formally Google Refine) [53], a free, open-source software for cleaning, normalizing, and transforming data.

### 2.3. Statistical, Bibliometric Analyses, and Systematic Review of Some Emerging Applications

Statistical analysis was used to investigate the trend of publication growth over the years. Further, bibliometric analyses, comprising of network maps of co-occurrence of article keywords, co-citation between articles, co-citation between scientific journals, and co-authorship between countries, were undertaken. These were deployed to identify the main research interests and directions, citations pattern, influential outlets for scientific works, and scientific collaboration between institutions and countries. These analyses were done in a free, open-source science mapping software, VOSviewer 1.6.16 [46]. It provides a distance-based map, wherein the relationship between items (i.e., journal, author, country, etc.) is reflected by the length of the distance between them. A shorter distance between two items indicates a strong relationship between them. Furthermore, each item of analysis is represented by a node (circle or rectangle), with a larger node indicating higher importance. The importance of an item may be assessed based on several documents, citations, average citations, etc. Items with the same color belong to the same cluster and, thus, indicate their co-citation of HEA/MCA research. Maps displayed, based on density visualization (e.g., for journals), indicate they receive a lot of citations or published more articles [46]. Finally, we conducted a systematic review of some emerging applications, based on the results of the bibliometric analyses and proposed potential future research directions.

## 3. Results

### 3.1. Trend of Scientific Publication in HEA/MCA, from 2004 to April 2021

**Figure 1:**
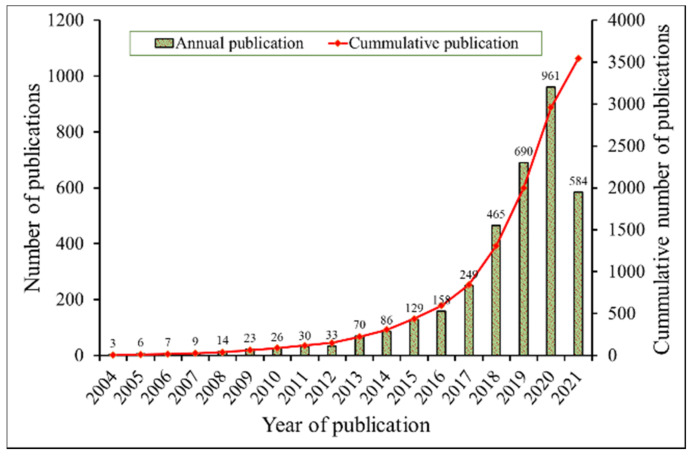
Trend analysis of HEA/MCA publications, between 2004 and 2021. Data were obtained from the Scopus database and plotted using Microsoft Excel.

Figure 1 shows the annual trend of scientific publication in HEA/MCA field, from 2004 until 25 April 2021. Cumulatively, 3541 research articles, based on HEA/MCA, have been published, up to the time of retrieving these data from the Scopus database. There has been a consistent annual growth in publication outputs over the years, since 2004. However, the annual output is less than 40 articles in the formative years of HEA/MCA (up until 2012). This may be ascribed to the misconception that multi-element alloys form glassy [1] or intermetallic compounds with complex crystal structures [2]. With the pioneering publications in this field [1,4], this wrong notion is dispelled, and many researchers begin to show interest in it, as indicated by the steepness of both annual and cumulative publications. It is expected that this increase will continue in the following years, as more developmental alloys are discovered and application areas are explored.

### 3.2. HEA/MCA Research Outlets

**Figure 2:**
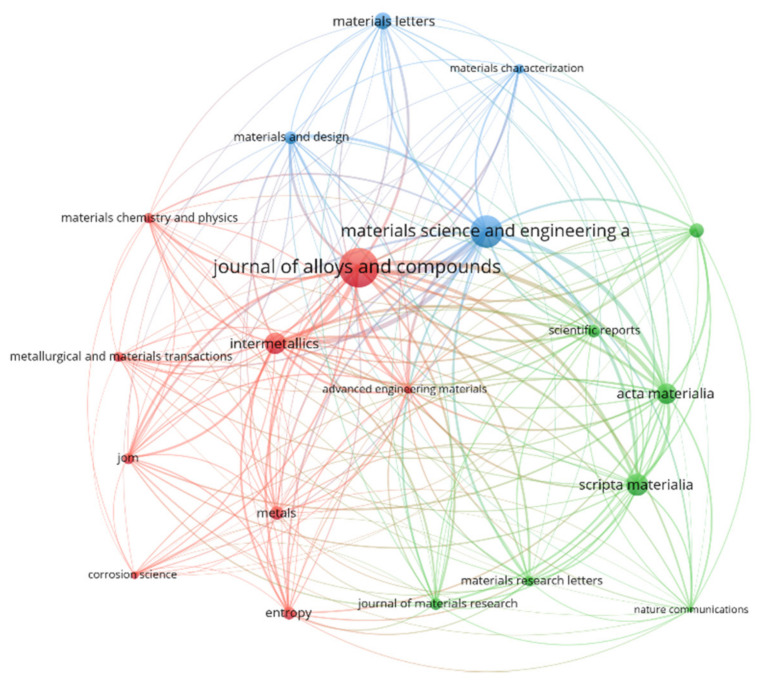
Network map of the top twenty research journals for HEA/MCA works. The Figure was generated using the VOSviewer software. To avoid journal names overlapping with one another, a journal was omitted. The unlabeled “green” node belongs to *Scripta Materialia*.

Identification of major research outlets for HEA/MCA works is highly important. This will help readers, researchers, and policy makers/funders to focus on important sources for staying up-to-date with the development in this field. Additionally, researchers can easily find appropriate outlets for their works to reach a wider audience. Some studies have shown that journal reputation enhances article citation [54,55]. Figure 2 shows the document network map of the top twenty research journals for HEA/MCA works.

Overall, 379 unique research journals have published works in this field. To generate the network map in Figure 2, the minimum number of documents of a source (i.e., journal) and minimum number of citations of a document (article) are set to 5 and 50, respectively. Although there are no standardized criteria for generating this type of network map in the published literature [56], we have chosen these criteria to prune the number of scientific sources (379) to a manageable size. Eighty-five of these journals meet these criteria, but the statistics and network map of only the top 20 (ranked on the number of publications) are shown in Table 1 and Figure 2, respectively.

In Figure 2, the journals are clustered into three groups, indicated by red (Cluster 1, with 9 journals), green (Cluster 2, with 7 journals), and blue (Cluster 3, with 4 journals). Journals in the same cluster signify higher co-citation between them. Furthermore, the larger the size of a node associated with a journal, the more influential/important it is, based on a number of articles published or total citations received. Finally, the shorter the length of the line between two journals, the stronger the relationship between them [46]. In Table 1, the *Journal of Alloys and Compounds* ranks first, with the highest total link strength (5217), number of publications (387), and citations (9432). The journal has published a staggering 11% of the whole of HEA publications, between 2004 and 2021. This highlights its importance as the most popular outlet for HEA/MCA research works. The next-ranked journals, in descending order of the number of publications, are *Materials Science* and *Engineering A, Scripta Materialia, Intermetallics*, and *Acta Materialia*. Put together, these five journals have published a combined 72% of all HEA/MCA works. Although some highly cited articles are not published in the top 20 list in Table 1, we have identified such articles, their sources, and highlighted their impacts under Section 3.4.

### 3.3. Scientific Collaboration Networks

#### 3.3.1. Co-Authorship of Researchers

Scientific collaboration between researchers facilitates knowledge exchange, cross-fertilization of ideas/concepts, and access to specialized research facilities. Furthermore, it has been used as one of the indices for ranking universities [44]. Therefore, it is imperative to assess the scientific collaboration between researchers in the field of HEA/MCA. Table 2 shows the results of the co-authorship of the top 20 authors, based on total link strength. The results were generated by setting the “minimum number of documents of an author to 5” and “minimum number of citations of an author to 10”. Of the 6545 researchers in the database, 927 satisfy these conditions. However, only the top 20 are shown in Table 2.

The five most prolific authors are Zhang Y., Liaw P.K., Liu Y., Wang L., and Li J. with 157, 149, 132, 104, and 102 published articles, respectively. A different ranking is obtained when total citations is used as a ranking basis. Thus, the first five, in descending order of total citations, are Yeh J.-W. (15899), Chen S.-K. (8362), Shun T.-T (6952), Liaw P.K. (6886), and Lin S.-J. (6472). Compared with the ranking of the most prolific authors, only Liaw P. K. appears in the top five most-cited authors. Therefore, it may be inferred that prolificity in article publication does not necessarily translate to more citations. High citation counts are more likely to be associated with the quality of research work, novelty, reputation of publishing journal, and journal publication model (open access or subscription-based) [54,55]. Similarly, cooperating institutions may also contribute to high citation counts, as indicated by the different clusters in Table 2. Other than Cluster 5, all the other clusters have at least two different collaborating institutions. While inter-institutional collaboration is apparent from the list in Table 2; however, it is instructive to note that inter-country collaboration is abysmally low among these top 20 authors, except for Cluster 2. This low level of inter-country scientific collaboration may be attributed to a lack of funding for international collaboration, political restriction on materials and data sharing, and differences in academic standards [57].

Furthermore, Yeh J.-W has about twice the total citations of the second-most cited researcher in Table 2. This may not be unconnected to his being one of the pioneers in this field, and his first early works of more than 15 years ago continue to receive citations in newer publications. To correct for the bias that older documents have had more time to acquire citations than recently published ones, authors are re-ranked based on normalized citations (norm. citations) [58]. Therefore, the top five are now Liaw P.K. (norm. citation 268.67), Zhang Y. (201.81), Li J. (150.29), Liu Y. (146.40), and Liu X. (128.15).

#### 3.3.2. Co-Authorship between Institutions and Countries

Bibliometric analysis of institutional collaboration is very useful for assessing the strength of research collaboration between institutions and/or countries. It also furnishes industrial partners with the necessary information to quickly locate prestigious institutions for establishing collaborative research and hastening the lab-to-market time of innovative products. To generate the results here, the “minimum number of documents of an institution” and “minimum number of citations of an institution” are set to 5 and 10, respectively. Of the 5730 organizations retrieved, 249 satisfy this threshold, but only the top 15 are selected for analysis and shown in Table 3.

The results in Table 3, below, show that the University of Tennessee, Knoxville (UoTK, United States) and University of Science and Technology Beijing, Beijing (USTB, China) are the foremost institutions in the field of HEA/MCA, in terms of the number of published research articles and total citations received. However, the total link strength of UoTK doubles that of USTB. The total link strength is an attribute that indicates the strength of co-authorship of a researcher (or institutions) with others [58]. Similarly, other indices, such as the number of research publications and total citations, are nearly double that of USTB, highlighting the leading role of UoTK. This is despite the fact that ground-breaking HEA/MCA research articles are published by researchers from National Tsing Hua University, Hsinchu (NTHU, Taiwan) Taiwan [2,4,13,59] and the UK [1]. However, the second-highest total citations are credited to NTHU (Taiwan), the institution of Prof. J.-W Yeh’s group, one of the first two research groups to publish articles on HEA. It is also important to note that the total link strength of NTHU is 6, indicating a low level of collaboration with other institutions. Likewise, of the 15 institutions listed in Table 3, seven are in China, based on the total *number of research articles*.

**Figure 3:**
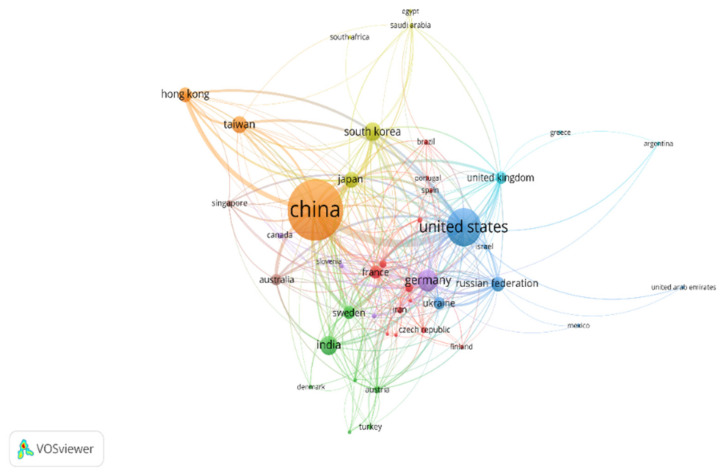
Scientific collaboration network of countries on HEA/MCA research. The Figure was generated using the VOSviewer software.

Other than inter-institutional collaboration, inter-country scientific collaborations enhance knowledge sharing and technology transfer. Figure 3 shows the active countries by the number of publications in the field of HEA/MCA. The figure is generated in VOSviewer by selecting co-authorship and countries as the type of analysis and unit of analysis, respectively. Further, the “minimum number of documents of a country” is set to 5, while the “minimum number of citations of a country” is set to 10. Of the seventy-two countries retrieved, 44 meet this threshold and are shown in Figure 3. The biggest node is associated with China, followed closely by the United States and Germany. The size of the nodes indicates the number of scientific articles published.

China is the most prolific country with 1643 publications, 32169 total citations, and 793 link strength. Following next is the United States, with 752 scientific publications and 28201 and 634 citations and total link strength, respectively. A closer inspection shows that China has published more than twice the number of HEA/MCA scientific articles emanating from the US. However, the total citations received by the US are almost on par with that of China. This may likely indicate a better quality of research emanating from the US. Other highly active countries, in descending order of the number of research publications, are Germany (299), India (243), South Korea (225), Taiwan (191), Japan (174), Russia (155), Hong Kong (153), and Sweden (126).

### 3.4. Citations Analysis of HEA/MCA Publications

**Figure 4:**
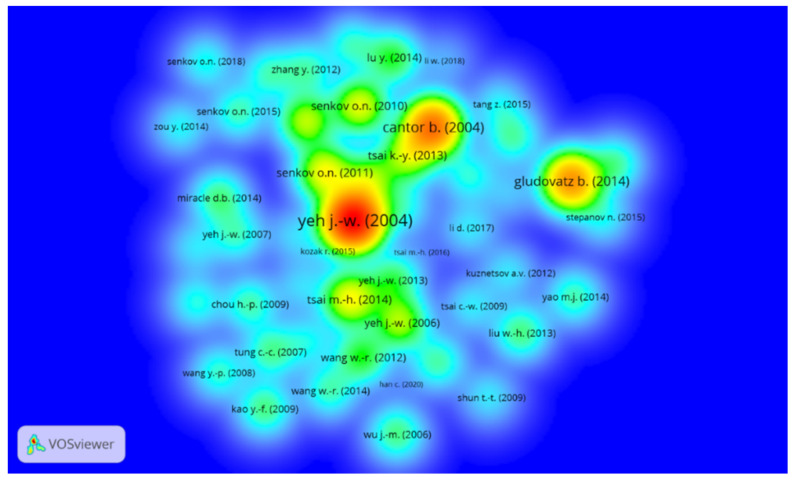
Network density map of most influential HEA/MCA research articles. The Figure was generated using the VOSviewer software.

This section presents the results of the analysis of the most influential publications. The minimum number of citations of a document was set to 20, with 925 of the 3543 articles meeting this criterion. To manage this large number of articles, only the top 50, based on the number of citations, are shown in Figure 4, while the top 10 most-cited articles are listed in Table 4, below. It is apparent from Figure 4 that the pioneering publications of Yeh J.-W et al. (2004) [4] and Cantor et al. (2004) [1] are the most influential articles in the HEA/MCA field. These two articles have combined total citations of 6899, which exceeds the total citations of the next six most influential articles. All these top 10 articles were published between 2004 and 2014, with only two being review articles [12,60]. To annul the comparative advantage of older articles having had more time to be cited, the articles are re-ranked, based on normalized citations (norm. citation). Normalized citations are calculated as the number of citations received by an article, divided by the average number of citations of all articles published in the same year [58]. Consequently, the top five most influential articles are Gludovatz B. et al. (2014), Zhang W. et al. (2018), Tsai K.-Y. et al. (2013), Senkov O.N. et al. (2018), and Senkov O.N. et al. (2011) [7,61,62,63]. All these articles are less than ten years old and have mainly investigated the fundamentals of HEA/MCA.

The pioneering articles of Yeh et al. [4] and Cantor et al. [1] are fundamental to the advancement of knowledge in the field of HEAs. In particular, Prof. Yeh’s group in Taiwan christened the high-entropy alloy materials and showed that simple melting and casting techniques can be used to produce them. Further efforts utilized spray coating [13], splat quenching [2], and sputtering techniques [59] to synthesize HEAs with outstanding mechanical, high oxidation, and corrosion-resistant properties [2,13,59]. These foundational experimental research efforts dispelled the long-held misconception about the development of complex, intermetallic compounds in multi-element-based alloys and provided the foundation and impetus for exploring a completely new set of strategies for developing new alloys. It is, therefore, on this note that many scientists have embraced this field and explored the potentials of these intriguing materials, as indicated in Figure 1.

From Table 4, the third most-cited HEA article “*A fracture-resistant high-entropy alloy for cryogenic applications*” was published in *Science* [7]. Although *Science* does not feature in the ranking of the 20 most influential journals publishing HEA works (see Table 1), the work of [7] has received more than 2000 citations. Of course, there may be some other excellent works in the scientific literature that may have not been covered in this review, owing largely to our selected inclusion/exclusion criteria. Many prior publications on HEAs focus on room-temperature mechanical characterization. However, Gludovatz and co-workers characterized the cryogenic mechanical properties of FeCoCrMnNi Cantor HEA and reported that its tensile strength, ductility, and fracture toughness are enhanced at cryogenic temperatures. On the contrary, many traditional alloys exhibit an inverse behavior. Although austenitic stainless (304 L and 316 L) and cryogenic (5 Ni and 9 Ni) steels have been reported to exhibit enhanced tensile strength and ductility, with decreasing temperature up to 77 K, their fracture toughness invariably decreases. The outstanding properties exhibited by FeCoCrMnNi HEA, which are attributed to room-temperature planar-slip dislocation transition to mechanical nanotwinning at cryogenic temperature, indicate their potential for cryogenic applications.

Among the most influential articles in Table 4, two are review articles from Prof. Yeh’s group [12,60]. Together, these articles have received about 1700 citations and discuss the fundamental principles of HEA formation and their properties. For instance, the authors proposed four core effects that are crucial to rationalizing the properties of HEAs. These include entropy effects (i.e., high configurational entropy of mixing stabilizes solid-solution at the detriment of intermetallic compounds), sluggish diffusion (i.e., slow diffusion and phase transformation kinetics, contributing to structural stability, high-temperature strength, etc.), severe lattice distortion (the presence of different atomic species, thus, increases strain energy, impedes dislocation movement, enhances solid-solution strengthening, etc.), and cocktail effects (a broad perspective on HEA properties, resulting from synergistic interactions among all the constituting elements).

Another influential article is “*Phase stability in high entropy alloys: formation of solid-solution phase or amorphous phase*”, published in *Progress in Natural Science: Materials International* [64]. The article has accumulated about 800 citations and highlights some important fundamental parameters to understanding solid-solution phase stability in HEAs. An earlier work by Zhang et al. [65] proposed the stability of solid-solution phases in HEA, based on the criteria of high ∆S_mix_, −15 kJ mol^−1^ ≤ ∆H_mix_ ≤ 5 kJ mol^−1^ and 1 ≤ δ ≤ 6, where ∆S_mix_, ∆H_mix_, and δ are change in mixing configurational entropy, mixing enthalpy, and atomic size difference, respectively. Guo et al. [64] extended this work to include the data on equimolar bulk metallic glasses and study two more parameters (electronegativity difference, Δχ and valence electron concentration, VEC). They proposed the following criteria: 0 ≤ δ ≤ 8.5, −22 ≤ ∆H_mix_ ≤ 7 kJ mol^−1^ and 11 ≤ ∆S_mix_ ≤ 19.5 J K^−1^ mol^−1^. Additionally, they show that Δχ and VEC do not have a significant effect on the stability of solid-solution phase in HEA. However, in one of their earlier studies, the authors highlight the importance of VEC in predicting the formation of either FCC or BCC-type solid-solution in HEAs [66]. Another major contribution from the research work “*Phase stability in high-entropy alloys: formation of solid-solution phase or amorphous phase*” [64] is that a high ∆S_mix_ is not the sole factor that influences the formation of solid-solution in HEAs. Some recent works support this conclusion [67,68,69]. In all, [66] contributes to our understanding of phase stability in HEAs.

Many research efforts have concentrated on investigating HEAs derived from transition metals (Fe, Ni, Co, and Cu). With the objective of developing new high-temperature structural materials, Senkov et al. [8] reported a systematic study of the microstructure and hardness of two refractory metal-based HEAs (WNbMoTa and WNbMoTaV). This work is not only one of the most influential studies (with 928 citations) in the HEA field, but also among the first to study refractory HEAs. Ever since, more studies on refractory HEAs have emerged [19,70,71]. However, more research on the tensile ductility, fracture toughness, oxidation resistance, and creep strength of refractory HEAs is needed to fully compare them with existing high-temperature metallic alloys [11]. The need to research in this area is underscored by the potential applications of refractory HEAs in the nuclear and aerospace industries, as high-temperature structural materials and thermal protection units.

### 3.5. HEA/MCA Keywords Analysis

A co-occurrence analysis is a visualization or mapping of the important concepts associated with a specific research field. In addition to assisting authors in identifying critical research areas and knowledge gaps, this type of analysis also provides information on the interconnectivity between research topics and their associated research methodologies. Thus, at a glance, researchers can access the aforementioned information from a network map of co-occurrence of keywords [56]. The results presented here are based on the keywords provided by authors with their articles. These “author keywords” should not be confused with the “index keywords” generated by Scopus, which may, sometimes, be different from “author keywords”.

#### Co-Occurrence Network of HEA/MCA Author Keywords

**Figure 5:**
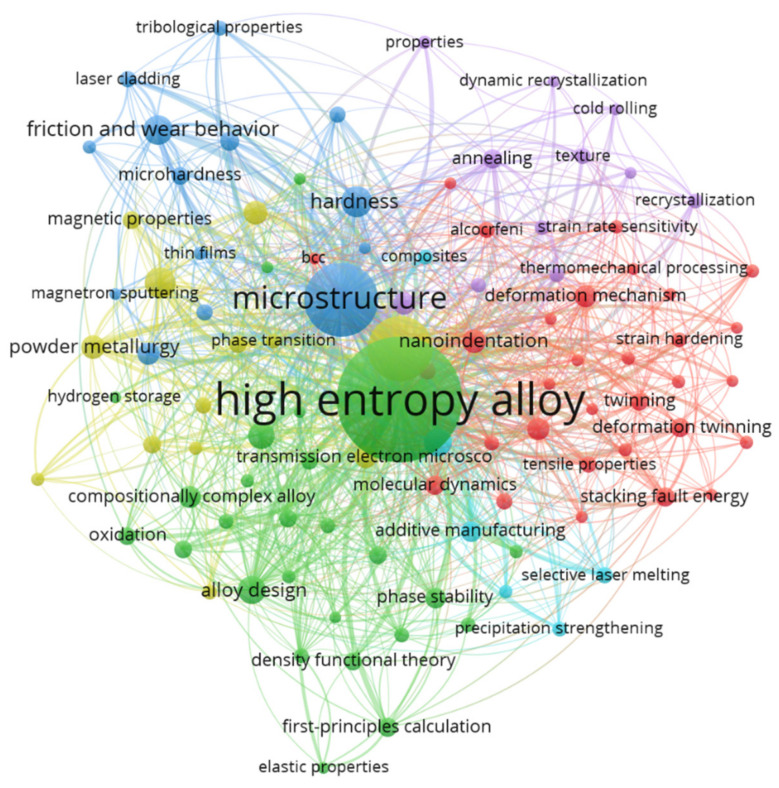
Co-occurrence network of keywords based on occurrences. Figure was generated using the VOSviewer software.

Figure 5 shows the co-occurrence network map of author keywords in the HEA/MCA field, based on the frequency of occurrence. The minimum number of occurrences of a keyword is 20, from which 101 keywords satisfy this threshold, out of 3594 author keywords.

**Figure 6:**
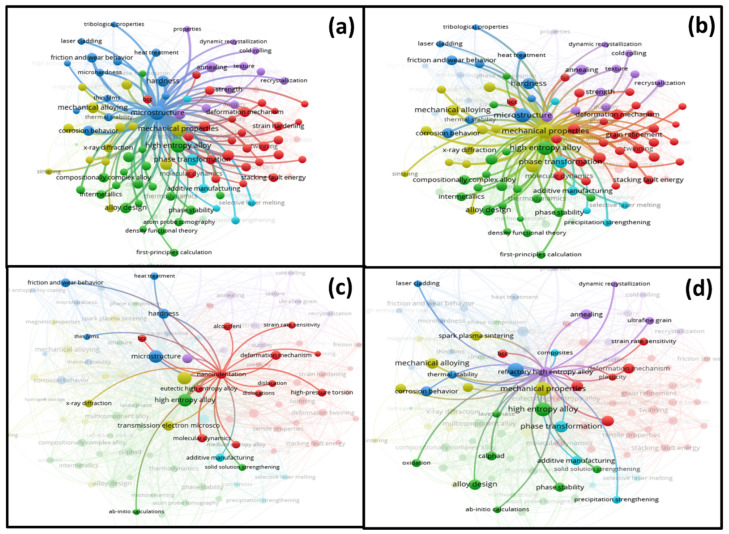
Co-occurring networks showing the links of other keywords frequently occurring with the keyword (**a**) “microstructure”; (**b**) “mechanical properties”; (**c**) “nanoindentation”; (**d**) “refractory high-entropy alloy”. All figures were generated using the VOSviewer, software.

Each node in Figure 5 represents a keyword, while the size of a node indicates the number of articles in which the keyword is provided. Furthermore, the thickness of a line connecting two or more keywords denotes the frequency of their co-occurrences. All the 101 keywords are grouped into six clusters. The first cluster appears in green color, with “high-entropy alloy” being the most prominent (2222 occurrences, link strength of 3993, and connected with virtually all other keywords—99 links). The second-most prominent keyword is “microstructure” in the blue cluster. Similar to “high-entropy alloy”, it is connected to 98 other keywords and has a link strength and occurrence of 1873 and 775, respectively. Co-occurring keywords with “microstructure” are shown in Figure 6a. The figure indicates that “microstructure” is frequently investigated, alongside powder processing techniques (e.g., “mechanical alloying”, “sintering”, etc.), mechanical characterization (e.g., “mechanical properties”, “hardness”, “friction and wear behavior”, etc.), and phase changes (e.g., “phase transformation”, “phase composition”, etc.), to mention but a few.

In the yellow cluster, the dominant keyword is “mechanical properties”. This keyword has been used 598 times, with a total link strength of 1488. The cyan cluster has “phase transformation” as the most important keyword (links = 63, link strength = 278, and occurrences = 118). The largest cluster (red) has 31 different keywords. They include “deformation mechanism”, “cantor alloy”, “nanoindentation”, “grain refinement”, “plastic deformation”, “fcc”, “bcc”, and “work hardening”. This cluster can be broadly grouped as a deformation/strengthening mechanism. The most influential keyword in this cluster is “nanoindentation”, with 47 links (i.e., co-occurring with other 47 keywords; see Figure 6c) and 81 occurrences in the reviewed literature. The last cluster is shown in purple and contains keywords such as “annealing”, “refractory high-entropy alloy”, “cold rolling”, “texture”, etc. This cluster broadly describes research interests involving ductility, strength enhancement, and high-temperature properties. “Refractory high-entropy alloy” has the highest link strength (156) and co-occurs with 47 other keywords (see Figure 6d). Since refractory high-entropy alloys exhibit properties like high melting points, resistance to oxidation, and high strength, the most economical synthesis route is via powder processing. Hence, it co-occurs with other keywords like “mechanical alloying”, “powder metallurgy”, “bcc”, “oxidation”, and “spark plasma sintering”.

### 3.6. Research Gaps and Potential Future Directions

Analysis of all the author-provided keywords in Figure 6 indicate that “hydrogen storage”, “machine learning”, “magnetron sputtering”, “high-entropy alloy coating”, and “dynamic recrystallization” have the lowest total link strengths of 20, 36, 37, 44, and 44, respectively. Besides, these keywords have fewer than 17 links, with the other 3500-plus keywords. This is an indication that they have not been extensively investigated by the scientific community. For instance, “hydrogen storage” (7 links) is an important topic of research interest in the development of cleaner energy storage and production, as well as reducing the deleterious impact of fossil fuels on the environment. Existing materials include metal-hydrides [72,73], graphene [74], carbon nanotubes [75], etc. However, most of these materials are deficient in serving as competitive hydrogen storage materials. Given the promising results and properties exhibited by HEA/MCA, especially their BCC structure and strained lattice, which can accommodate large volumes of hydrogen [76], hydrogen production, and storage, may find a unique niche in this field. Most of the existing studies typically utilize Ti, V, Nb, Hf, Zr, Cr, Mn, Fe, Ni, and Mg [76,77,78]. Other elemental combinations may also be investigated for their hydrogen storage capacities.

- “Machine learning” is another keyword that requires further and elaborate investigation from researchers in this field. Its total link strength and link of 36 and 15, respectively, indicate that it has not been fully explored. Machine learning (ML) belongs to the soft computing method that can be used to optimize the synthesis of HEA/MCA, predict phases, properties, and minimize expensive and time-intensive experimentations [79,80]. This approach has been successfully deployed to predict the particle sizes of mechanically milled, magnesium-based metal powder [81] and discover novel active layers in organic solar cells [82], light-emitting diodes [83], and nickel superalloys [84]. Furthermore, ML tools can be conveniently deployed to extract greater and more accurate information from synthesis conditions and parameters [85]. Nonetheless, some recent works on HEA/MCA, applying the ML approach, have appeared in the literature [80,86,87]. More efforts are, however, needed in this direction. HEA/MCA can also benefit from this technique to accelerate the discovery, development, and deployment of novel HEA/MCA materials.
- Another keyword that is less explored is “tribological properties” (32 occurrences), with 14 links with other keywords. A decent number of articles have been published with the keyword “friction and wear behavior” (45 links and 129 occurrences) under dry conditions. However, only a handful of research has emerged in the HEA/MCA literature discussing the effect of lubrication [88,89]. Many industrial processes involve metallic parts that are in motion, relative to one another, where lubricants are used to minimize wear and friction. For practical purposes, it is imperative to investigate the tribological properties and understand the friction and wear mechanisms of HEA parts under lubricating conditions. This is an important research gap that requires research attention. Some other functional application areas that need further and in-depth research include catalysis (8 occurrences), superconductivity (13 occurrences), and thermo-electricity (14 occurrences). Although some researchers have reported some results in HEA/MCA catalysis [32,33,34,90], superconductivity [35,36], and thermo-electric properties [39], research in these areas is still exploratory and fairly recent. These application areas are important, and HEA/MCA materials are good contenders to perform very well.
- An important research area, with a huge potential to generate important discoveries, is developing composites of HEA to enhance their properties. According to the analysis of the keyword “composites”, it is rarely used, as it co-occurred with only 23 other keywords and appears about 34 times in the surveyed literature. Composite development can be employed to improve the properties of HEA, such as hardness/wear [91,92], tensile strength [93], and application as binders in cermets [94]. More studies are required to unravel the effect of different reinforcement materials and their phases on the properties of HEA/MCA composite materials.

## 4. Systematic Review of Emerging Application Areas

**Figure 7:**
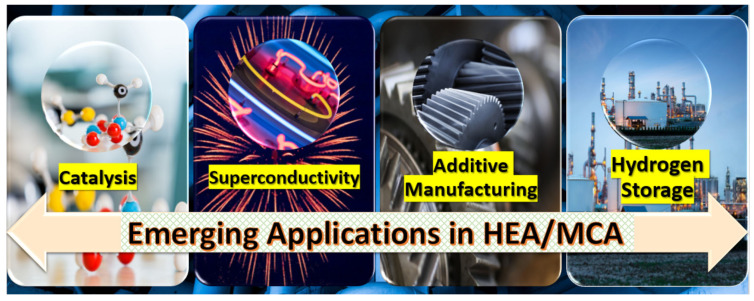
Emerging application areas in the field of HEA/MCA. All representative images were taken from Microsoft Office 365.

Other than the less-explored areas identified in the previous section, a brief review of four important emerging application areas are given in this section. These include hydrogen storage, additive manufacturing, catalysis, and superconductivity, as illustrated in Figure 7.

### 4.1. Hydrogen Storage

Hydrogen storage (HS) is of immense research importance in the development of cleaner energy, as an alternative to fossil fuels, as well as reducing their impact on the environment. However, its safe and efficient storage remains a huge challenge. HS materials are expected to have high storage capacity, low decomposition temperature and pressure for the release of hydrogen gas, low cost, readily available, light weight, and high cyclability [95,96]. Conventionally, storage techniques include the gas- (e.g., use of high-pressure gas cylinders) and liquid-phases (e.g., open cryogenic tanks [96]). However, these techniques pose some technical challenges, including the risk of explosion, an energy-intensive liquefaction process, etc. [96]. Solid storage (e.g., metal/alloy-hydrides [72,73]) is regarded as safer and more convenient, compared with the previous two techniques. Some metals (e.g., Li, Be, Na, Mg, B, and Al) and alloys (e.g., Mg/Ni, Mg/Cu, etc.) exhibit a high affinity to chemisorb hydrogen at low temperatures to yield metal/alloy-hydrides, while the hydrogen can be released at high temperatures. More so, metal/alloy hydrides have theoretically higher hydrogen storage capacity than the liquid or gaseous state [97]. However, experimental results show that many metal-hydrides exhibit a low gravimetric density of absorbed hydrogen [96] and require high decomposition temperature, thus precluding them for deployment in batteries or fuel cells [95]. To meet commercial uses, HS materials are required to exhibit a minimum of 6.5 wt.% and 65 g/L hydrogen, within the decomposition range of 333 and 393 K [97]. Therefore, recent research efforts have explored HEAs as potential HS materials.

HEAs are characterized by severe lattice distortion, which is hypothesized to create more adsorption sites, such as vacancies and dislocations, for hydrogen atoms [76,98]. Therefore, some reports have examined the performance of some HEAs for HS application [99,100]. A general strategy is to include at least one excellent hydride-forming element as one of the HEA constituents, synthesize the HEA in either the BCC [76,101] or C-14 Laves phase [77,102], and then transform it to the FCC phase, during hydrogen adsorption, to form HEA-hydride. HS in HEAs usually requires a high-temperature activation step [98,99]. A systematic study by Edalati et al. [77] shows that TiZrNbHfTa HEA can be engineered for room temperature, reversible hydrogen storage if its VEC = 6.4. A similar study is reported by Zhang et al. [98].

In the surveyed HEA/MCA literature, arc melting is the preferred synthesis method, followed by mechanical milling before the hydrogenation reaction [76,100,101]. This added step (i.e., mechanical milling) may lead to powder contamination and inadvertently complicates the synthesis method. Laser-engineered net shaping (LENS) [99,102] is another method that has been investigated. While it can fabricate net shape samples, laser melting of alloy or elemental powder also leads to elemental segregation and chemical inhomogeneity ([99]). A few studies have also studied the application of high-energy ball milling/mechanical alloying as a synthesis method for lightweight hydrogen storage HEA incorporating Mg [103,104,105].

This is a developing field, and some technical challenges must be overcome to maximize the potential of HEAs for HS applications. (i) The reported values of hydrogen/metal (H/M in wt.%) gravimetric capacity are still low (1.78% [100], 1.81% [99], 2.5% [76], etc., as compared with the 13% in high pressure gas cylinder [96]. (ii) The highest HEA H/M gravimetric ratio reported, to date, is 2.5% at 573 K [76]. There is a need to develop HEAs that can attain room temperature activation and dehydrogenation [77]; (iii) Most reports characterize the synthesized HEAs in powder form. Powder handling comes with its own challenges, including safety issues, packing, and sintering after long-term cycling. Therefore, future research efforts may investigate HS properties of bulk and nanoporous HEAs. (iv) The cycling capacity of HEAs requires enhancement and stability to overcome the deterioration of HS capacity, with increasing hydrogenation cycles [98].

### 4.2. Additive Manufacturing

Conventionally, a vast majority of HEA/MCA has been fabricated by different casting techniques (e.g., arc melting or induction melting). Nonetheless, casting has some disadvantages, which include various casting defects (e.g., shrinkages and pores [106]), susceptibility of elements with low melting points (e.g., Mg and Zn) to evaporate (thus, resulting in compositional inhomogeneity [30]), and limitation in producing intricate shapes [107]. Consequently, alternative fabrication techniques, such as additive manufacturing (AM), have emerged. AM is a layer-by-layer deposition of a metal or alloy powder that conforms to a computer-aided design (CAD) model of a 3D object. A high-energy source, such as a laser, plasma, or electron beam, is then used to fuse the deposited layers into a solid object [106]. Therefore, AM has high design freedom, can be used to fabricate functionally graded objects, and offers higher heating and cooling rates [108].

There are three broad categorizations of the AM techniques: powder bed systems, powder feed systems, and binder-based systems [109]. Another categorization is based on the energy source, i.e., laser (e.g., direct laser deposition (DLD) and selective laser melting/laser beam melting (SLM/LBM)) and electron beam (e.g., selective electron beam melting (SEBM)), which are already covered in some review works [106,110]. Pioneering works in AM/HEA/MCA focus on HEA coatings made by laser cladding [111,112], while bulk samples are reported in later publications [99,113,114,115]. Most of the reports in this niche are biased towards Cantor-based alloys [111,113,114,116,117], while a few have investigated refractory HEAs [99,118,119,120,121].

From the microstructural perspective, AM-manufactured HEA/MCA are similar to their cast counterparts [110,113,122]. This is not surprising, as both methods rely on melting and rapid solidification. However, AM HEAs tend to exhibit finer grain sizes, due to more rapid cooling rates, compared with cast HEA of similar composition [113,122]. Therefore, AM HEAs possess improved mechanical properties [113,122,123], compared with cast HEAs. Various crystalline microstructures of solid-solution (e.g., FCC [122], BCC [24], and FCC+ BCC [123]) and intermetallic [99] phases have been reported.

Despite the attractiveness of the AM techniques, some key challenges have also emerged. These include the requirement of high-purity powders (pre-alloyed or elemental mix) with specific characteristics and need to recycle powder to partially offset its high cost [110]. Besides, elemental segregation can occur when working with powders containing elements with different melting points. Process optimization is also required to minimize manufacturing defects (e.g., cracks [106]). Further, the mechanical properties investigated are largely limited to tensile tests, and more characterizations on fatigue and creep behaviors are required [106,107]. Due to the poor surface finish of most AM HEA samples, post-machining is required, which may add to the production cost [107]. All these challenges represent potential future research directions. Other future outlooks may include the deployment of high-throughput synthesis techniques to fabricate 3D samples with varied compositions for rapid alloy screening [118,120]. This may be combined with machine learning techniques [124] or thermodynamic modeling [110,117].

### 4.3. Catalysis

A great deal of research effort has been devoted to the synthesis and mechanical characterization of HEA/MCAs [1,17,18,28,125]. Catalytic properties are only recently being investigated [32,34,126,127,128,129,130]. The synergistic chemical reactivity of the individual elements in a HEA/MCA, which may be designed to comprise catalytically active metals, as well as its severe lattice distortion, are believed to significantly enhance the chemical reactivity of HEA/MCA [128,131]. Therefore, HEA/MCAs are particularly attractive in catalysis, as they have the potential to replace commonly used catalysts.

In this regard, HEA/MCAs have been developed from low-cost, non-noble metals, such as Al, Co, Fe, Cu, Zn, Cr, etc., [34,130,132], while others contain more expensive noble or platinum-group metals, such as platinum, palladium, and iridium [34,126,129]. Examples of the different catalytic applications investigated thus far include water splitting (together with hydrogen and oxygen evolution reactions) [6,126,127,132,133], methanol oxidation [134], degradation of azo dyes [128], carbon dioxide/carbon monoxide reduction reactions [129], and hydrogenation of p-nitrophenol [130].

HEA/MCA catalysts have been synthesized via powder metallurgy [6,128], melt processing techniques [133], and a combination of both techniques [129]. The catalysts exist as free-standing, bulk structures [127,133] or powders dispersed in a binder matrix [34,129]. For enhanced catalysis, HEA catalysts with large surface areas have also been synthesized by various methods, such as the cryogrinding of melt-processed bulk [129], use of blowing agents [6,90], and chemical dealloying techniques [34,126].

This niche is still in its infancy; therefore, more research works are required to fully harness the potentials of HEA/MCAs. For instance, the hierarchical structure may be engineered into nanoporous HEAs for enhanced catalysis. Given the large number of HEA/MCAs, it will be experimentally impossible and expensive to investigate all of them. Therefore, computational approaches (e.g., density functional theory (DFT) and machine learning techniques [33,135]) may be deployed to accelerate the discovery and design of HEA/MCAs with enhanced catalytic activities.

### 4.4. Superconductivity

Another emerging application field in HEAs/MCAs is superconductivity (SC). SC is defined as “the process by which, below a certain characteristic critical/transition temperature, (*T_c_*), current-carrying electrons can move without hindrance (resistance) through a metal or metallic material” [136]. SC materials have many important applications, such as superconducting electromagnets (found in maglev trains), coils used in magnetic resonance imaging (MRI) and nuclear magnetic resonance (NMR) machines, beam and focusing components in particle accelerators, and NMR microscopes [137].

Most of the studies on SC utilize transition elements, such as Hf, Nb, Ta, Ti, and Zr, which have been shown to exhibit SC at ambient pressure [136]. The first study on HEA/MCA by Koželj et al. [36] utilizes the Ta-Nb-Hf-Zr-Ti system, which exhibits a type II SC property, with a transition temperature (*T_c_*) of about 7.3 K. Follow-up research by the same group investigates the same alloy system, but at different elemental concentrations and annealing heat treatment [35]. The authors show that both equimolar and non-equimolar concentrations of the Ta-Nb-Hf-Zr-Ti system exhibits SC, with *T_c_* varying between 5.0 and 7.3 K.

Furthermore, a HEA superconductor from the Ta-Nb-Hf-Zr-Ti system exhibits unusual zero-resistance superconductivity at extremely high pressures of up to 190.6 GPa, which is attributed to its highly stable crystal and electronic structures [138]. The *T_c_* is about 7.7 K at ambient pressure but shifts to a higher temperature as pressure is increased. This material may potentially be useful for applications requiring extreme pressures. There are also other reports on the Ta-Nb-Hf-Zr-Ti system [139,140]. Similarly, Stolze et al. [141] reported SC in the Zr-Nb-Mo-Re-Ru, Hf-Ta-W-Ir-Re, and Hf-Ta-W-Pt-Re systems. They show that *T_c_* is influenced by the lattice parameter (a) and valence electron count (VEC). Specifically, while *T_c_* increases linearly with VEC, it exhibits an inverse relationship with the lattice parameter. The phases in the reported SC HEA/MCAs can be broadly grouped into four, i.e., BCC [35,36,140], HCP [142,143], α-Mn [141], and CsCl [144].

As this is an emerging field, more research efforts are required to provide a fundamental understanding of the physics of superconducting HEAs. Furthermore, the FCC-type superconducting HEAs are yet to be reported. Future studies may investigate the existence, or otherwise, of this crystal class in SC HEA/MCAs. It is also important to develop HEAs with higher *T_c_*, as this can quickly catalyze their deployment in practical applications.

## 5. Limitations of Study

It is imperative to enumerate some of the possible limitations of this study, especially the dataset used for bibliometric analysis. Our dataset comes from only one database; therefore, there is the possibility of missing some relevant research articles that are not indexed in the Scopus database. Next, the dataset excludes articles published in languages other than English. Furthermore, other academic sources, such as conferences, books, encyclopedias, etc., have not been considered in our analysis.

## 6. Conclusions

This study provided a holistic bibliometric mapping of the HEA/MCA field by analyzing the existing literature, from 2004 to May 2021, using the VOSviewer software. More than 3500 research articles were analyzed. Although the first papers appeared in 2004, trend analysis showed that significant research interest in this field commenced in 2013. The most prolific authors were from China, USA, and Germany, with these countries having limited collaborations with other countries. Furthermore, *Journal of Alloys and Compounds*, *Materials Science and Engineering A*, *Scripta Materialia*, *Intermetallics*, and *Acta Materialia* were notable research outlets, with more than 100 published articles each. Based on the co-occurrence frequencies of these keywords, some research gaps and future research directions were proposed, which included the application of machine learning approaches for predicting and synthesizing HEA/MCA, compositing HEA/MCA for property enhancement, and investigating functional applications, such as hydrogen storage. In addition, a brief systematic review of some emerging application areas, covering hydrogen storage, additive manufacturing, catalysis, and superconductivity, was undertaken. Future studies may consider the inclusion of other academic sources and articles in other languages for similar bibliometric analyses. The results of this bibliometric study will support experts in improving their knowledge of the field and may spur them to develop new ideas.

## Figures and Tables

**Table 1:** Bibliometric analysis of the top twenty research journals for HEA/MCA works.

Rank	Journal	Number of Publications	Total Citations	Total Link Strength
1	Journal of Alloys and Compounds	387	9432	5217
2	Materials Science and Engineering A	279	8993	4529
3	Scripta Materialia	160	3296	2218
4	Intermetallics	149	6836	3455
5	Acta Materialia	139	6914	3122
6	Materials Letters	102	2062	966
7	Journal of Materials Science and Technology	82	818	1099
8	Metals	74	734	942
9	Entropy	71	1787	1157
10	Scientific Reports	70	2620	1198
11	Materials and Design	69	2462	1278
12	Journal of Materials Research	61	1142	1164
13	JOM	55	2883	1329
14	Materials Chemistry and Physics	52	1527	827
15	Materials Research Letters	52	2930	1166
16	Metallurgical and Materials Transactions A	48	1513	746
17	Materials Characterization	46	915	821
18	Advanced Engineering Materials	36	4934	1829
19	Corrosion Science	32	1181	493
20	Nature Communications	19	2708	796

**Table 2:** Co-authorship analysis of authors.

Rank	Author	Cluster	Institution/Country	Total Link Strength	Documents	Total Citations	Normalized Citations
1	Zhang Y.	2	University of Science and Technology, Beijing, China	205	157	6261	201.81
2	Liaw P.K.	2	University of Tennessee, Knoxville, United States	198	149	6886	268.67
3	Liu Y.	1	Tsinghua University, Beijing, China	189	132	2414	146.40
4	Li J.	3	Hunan University, Changsha, China	180	102	1654	150.29
5	Wang J.	3	Northwestern Polytechnical University, Xi’an, China	154	81	1240	95.58
6	Wang Z.	3	City University of Hong Kong, Kowloon, Hong Kong	131	83	1493	70.35
7	Wang L.	2	Harbin Institute of Technology, Harbin, China	129	104	1492	110.06
8	Wang Y.	1	University of Science and Technology Beijing, Beijing, China	125	80	1320	113.23
9	Liu X.	4	University of Science and Technology Beijing, Beijing, China	120	74	1470	128.15
10	Liu C.T.	3	City University of Hong Kong, Kowloon, Hong Kong	118	51	2929	86.86
11	Wang H.	4	Wuhan University of Technology, Wuhan, China	108	64	1936	112.10
12	Wu Y.	4	Shandong University, Jinan, China	107	59	1993	125.13
13	Wang T.	5	Dalian University of Technology, Dalian, China	106	58	2198	75.00
14	Liu B.	1	Central South University, Changsha, China	105	58	1185	72.76
15	Lu Y.	5	Dalian University of Technology, Dalian, China	99	54	2044	81.19
16	Kai J.J.	3	City University of Hong Kong, Kowloon, Hong Kong	93	34	519	40.07
17	Zhang H.	1	Tsinghua University, Beijing, China	85	65	1331	53.66
18	Wang X.	1	University of Science and Technology Beijing, China	80	52	533	36.97
19	Yang T.	3	Peking University, Beijing, China	77	30	836	48.28
20	Chen D.	3	City University of Hong Kong, Kowloon, Hong Kong	72	25	609	52.49

**Table 3:** Co-authorship analyses between the top fifteen institutions.

	Institution	Country	Number of Research Articles	Total Citations	Total Link Strength
1	University of Tennessee (Department of Materials Science and Engineering), Knoxville, United States	United States	137	5693	93
2	University of Science and Technology Beijing (State Key Laboratory for Advanced Metals and Materials), Beijing, China	China	78	3150	46
3	Central South University (State Key Laboratory of Powder Metallurgy), Changsha, China	China	62	749	22
4	Northwestern Polytechnical University (State Key Laboratory of Solidification Processing), Xi’an, China	China	50	878	7
5	Beijing Institute of Technology (School of Materials Science and Engineering), Beijing, China	China	42	532	10
6	Oak Ridge National Laboratory (Materials Science and Technology Division), Oak Ridge, United States	United States	33	1723	26
7	National Tsing Hua University, Hsinchu (Department of Materials Science and Engineering), Taiwan	Taiwan	31	3218	6
8	Dalian University of Technology (Key Laboratory of Solidification Control and Digital Preparation Technology), Dalian, China	China	28	927	8
9	Taiyuan University of Technology (College of Materials Science and Engineering), Taiyuan, China	China	21	245	20
10	Hunan University (State Key Laboratory of Advanced Design and Manufacturing for Vehicle Body), Changsha, China	China	20	184	19
11	Royal Institute of Technology (Department of Materials Science and Engineering), Stockholm, Sweden	Sweden	19	431	8
12	National Energy Technology Laboratory, Albany, United States	United States	18	1382	19
13	Taiyuan University of Technology (Key Laboratory of Interface Science and Engineering in Advanced Materials), Taiyuan, China	China	14	111	14
14	Argonne National Laboratory (X-ray Science Division), Argonne, United States	United States	13	368	17
15	Uppsala University (Department of Physics and Astronomy, Division of Materials Theory), Uppsala, Sweden	Sweden	11	294	8

**Table 4:** Top 10 most-cited articles in the field of HEA/MCA.

Rank	Authors	Title of Publication	Journal	Citations	Norm. Citations
1	Yeh J.-W. et al. (2004)	Nanostructured high-entropy alloys with multiple principal elements: novel alloy design concepts and outcomes	Advanced Engineering Materials	4409	1.86
2	Cantor B. et al. (2004)	Microstructural development in equiatomic multicomponent alloys	Materials Science and Engineering A	2490	1.05
3	Gludovatz B. et al. (2014)	A fracture-resistant high-entropy alloy for cryogenic applications	Science	2010	16.54
4	Tsai M.-H. et al. (2014)	High-entropy alloys: a critical review	Materials Research Letters	1036	8.53
5	Senkov O.N. et al. (2011)	Mechanical properties of Nb25Mo25Ta 25W25 and V20Nb20Mo20Ta20W20 refractory high entropy alloys	Intermetallics	1023	9.26
6	Senkov O.N. et al. (2010)	Refractory high-entropy alloys	Intermetallics	928	7.77
7	Tsai K.-Y. et al. (2013)	Sluggish diffusion in Co-Cr-Fe-Mn-Ni high-entropy alloys	Acta Materialia	815	10.06
8	Guo S. and Liu C. T. (2011)	Phase stability in high entropy alloys: formation of solid-solution phase or amorphous phase	Progress in Natural Science: Materials International	788	7.14
9	Yeh J.-W. (2006)	Recent progress in high-entropy alloys	Annales de Chimie Science des Matériaux	748	3.30
10	Tong C.-J. et al. (2005)	Microstructure characterization of AlxCoCrCuFeNi high-entropy alloy system with multiprincipal elements	Metallurgical and Materials Transactions A	736	3.85

## Data Availability

Data were obtained from the Scopus database and available at https://www.scopus.com (accessed on 3 January 2022).
